# Development and validation for prognostic nomogram of epithelial ovarian cancer recurrence based on circulating tumor cells and epithelial–mesenchymal transition

**DOI:** 10.1038/s41598-021-86122-4

**Published:** 2021-03-22

**Authors:** Jiani Yang, Jun Ma, Yue Jin, Shanshan Cheng, Shan Huang, Nan Zhang, Yu Wang

**Affiliations:** Department of Obstetrics and Gynecology, Renji Hospital, School of Medicine, Shanghai Jiaotong University, Shanghai, China

**Keywords:** Cancer, Biomarkers

## Abstract

We aimed to determine the prognosis value of circulating tumor cells (CTCs) undergoing epithelial–mesenchymal transition in epithelial ovarian cancer (EOC) recurrence. We used CanPatrol CTC-enrichment technique to detect CTCs from blood samples and classify subpopulations into epithelial, mesenchymal, and hybrids. To construct nomogram, prognostic factors were selected by Cox regression analysis. Risk stratification was performed through Kaplan–Meier analysis among the training group (n = 114) and validation group (n = 38). By regression screening, both CTC counts (HR 1.187; 95% CI 1.098–1.752; *p* = 0.012) and M-CTC (HR 1.098; 95% CI 1.047–1.320; *p* = 0.009) were demonstrated as independent factors for recurrence. Other variables including pathological grade, FIGO stage, lymph node metastasis, ascites, and CA-125 were also selected (*p* < 0.005) to construct nomogram. The C-index of internal and external validation for nomogram was 0.913 and 0.874. We found significant predictive values for the nomogram with/without CTCs (AUC 0.8705 and 0.8097). Taking CTC counts and M-CTC into separation, the values were 0.8075 and 0.8262. Finally, survival curves of risk stratification based on CTC counts (*p* = 0.0241), M-CTC (*p* = 0.0107), and the nomogram (*p* = 0.0021) were drawn with significant differences. In conclusion, CTCs could serve as a novel factor for EOC prognosis. Nomogram model constructed by CTCs and other clinical parameters could predict EOC recurrence and perform risk stratification for clinical decision-making.

*Trial registration* Chinese Clinical Trial Registry, ChiCTR-DDD-16009601, October 25, 2016.

## Introduction

Ovarian cancer was a leading cause of death among gynecological cancers, with 21,750 new cases and 13,940 deaths estimated for 2020 in the United States^1^. Due to the lack of early symptoms and physical signs, over 70% of patients are diagnosed as advanced stages, resulting in a poor prognosis with a 10-year survival rate of 5–21%^2^. Despite development in treatment techniques, approximately 80% of epithelial ovarian cancer (EOC) patients will suffer cancer recurrence after the primary treatment of standard cytoreductive surgery followed by adjuvant platinum-based chemotherapy^3^.

Therefore, effective methods for predicting EOC prognosis are of clinical significance to improve survival. The circulating tumor cells (CTCs), originating from solid tumors, are related to the hematogenous metastasis of various carcinomas, such as breast, prostate, and ovarian cancer^4–6^. CTCs disseminate to distant sites through phenotypic changes, including epithelial–mesenchymal transition (EMT) that could help them to penetrate blood vessels^7^. In hepatocellular carcinoma, Qi et al.^8^ demonstrated that the epithelial-to-mesenchymal–CTC ratio was significantly associated with cancer recurrence and progression. Thus, apart from CTC counts, mesenchymal–CTC (M-CTC) percentage also has clinical relevance as a minimal-invasive approach to predict cancer recurrence and guide clinical therapy^8, 9^.

Recently, CTC detection and isolation based on physical properties have been applied in various solid tumors^6, 10, 11^. However, these approaches might fail to classify aggressive CTC subpopulations that undergo the EMT process. In this study, we used the CanPatrol CTC-enrichment technique based on the RNA in situ hybridization (RNA-ISH) to identify and classify all CTC subpopulations including epithelial (E-CTCs), mesenchymal (M-CTCs), and epithelial/mesenchymal hybrids (hybrid-CTCs) with high efficiency^12^. This technique has been used in a range of carcinomas to predict prognosis^8, 13^. However, to the best of our knowledge, this is the first prospective study to classify the prognosis value CTC of subpopulations undergoing EMT in EOC through the CanPatrol CTC-enrichment approach.

Moreover, given the poor prognosis of EOC, an effective risk stratification system is of great importance for clinicians in the therapeutic decision-making process^14^. So, we aimed to construct the nomogram, a comprehensive model with a graphical representation that could evaluate the numerical probability of cancer recurrence for individual^15^. Most previous prognosis models were constructed based on general factors such as clinical stage, pathological grade, tumor histology, and CA-125, with limited predictive value^16, 17^. So, the objective of our prospective study was to construct and validate the prognosis nomogram based on CTCs, more accurately as compared to current models in practice. By using this nomogram for risk stratification, we hope to develop a prediction tool, which could support therapeutic decision-making and might consequently improve the prognosis of EOC patients.

## Results

### Clinicopathological characteristics

Demographic and clinicopathological features of all the EOC patients, including the training group (n = 114) and validated group (n = 38), were listed in Table 1 and Table 2. Patients with early-stage (FIGO I or II) and advanced stage (FIGO III or IV) accounted for 39 (25.66%) and 113 (74.34%), respectively. Patients diagnosed as low pathological grade (G1–G2) and high pathological grade (G3) accounted for 51 (33.55%) and 101 (66.45%) of all patients involved. There were 72 (47.37%) patients with histology-proved lymph node metastasis, and 58 (38.16%) cases presented with ascites. The mean value (± SD) of CTC counts in 5 mL of blood, M-CTC percentage, E-CTC percentage, and hybrids-CTC percentage were 8.70 ± 5.69, 0.24 ± 0.19, 0.57 ± 0.25, and 0.19 ± 0.11, respectively. The mean value (± SD) of CA-125 was 990.71 ± 365.41 (U/mL).The median time of follow-up was 30 months (range 24–35 months). The investigated clinical data of patients from both groups were analyzed and no significant difference was found, indicating no selection bias (*p*-value ≥ 0.05). Moreover, we also involved 30 patients diagnosed with benign gynecologic diseases as controls, with the mean age of 57.98 ± 9.53. Among the control group, the mean value (± SD) of CTC counts in 5 mL of blood, M-CTC percentage, and CA-125 were 1.04 ± 0.73, 0.02 ± 0.08, and 14.26 ± 7.31 (U/mL), significantly lower compared to those EOC patients (*p*-value < 0.05).

**Table 1:** Baseline clinicopathological characteristics of the 152 epithelial ovarian cancer (EOC) patients involved grouped by training set and validation set.

Variables	Total (n = 152)	Training group (n = 114)	Validated group (n = 38)	*p*-value
Age (years)	58.06 ± 9.47	57.81 ± 10.25	58.10 ± 9.73	0.879
BMI (kg/m^2^)	22.86 ± 0.73	22.91 ± 0.47	22.85 ± 0.93	0.604
**Menopausal status, n (%)**				0.924
Pre/peri-menopause	65 (42.76%)	49 (32.24%)	16 (10.53%)	–
Post-menopause	87 (57.24%)	65 (42.76%)	22 (14.47%)	–
**Fertility history, n (%)**				0.852
0–1	82 (53.95%)	62 (40.79%)	20 (13.16%)	–
≥ 2	70 (46.05%)	52 (34.21%)	18 (11.84%)	–
Tumor size (cm)	7.26 ± 3.15	6.39 ± 4.62	7.37 ± 3.21	0.285
**Pathological grade, n (%)**				0.765
G1–2	51 (33.55%)	39 (25.66%)	12 (7.89%)	–
G3	101 (66.45%)	75 (49.34%)	26 (17.11%)	–
**FIGO stage, n (%)**				0.748
I–II	39 (25.66%)	30 (19.74%)	9 (5.92%)	–
III–IV	113 (74.34%)	84 (55.26%)	29 (19.08%)	–
Lymph node metastasis, n (%)	72 (47.37%)	52 (34.21%)	20 (13.16%)	0.453
Ascites, n (%)	58 (38.16%)	43 (28.29%)	15 (9.87%)	0.847
**Histological type, n (%)**				0.954
Serous	92 (60.53%)	69 (45.39%)	23 (15.13%)	–
Mucinous	24 (15.79%)	18 (11.84%)	6 (3.95%)	–
Endometrioid	17 (11.18%)	12 (7.89%)	5 (3.29%)	–
Others	19 (12.50%)	15 (9.87%)	4 (2.63%)	–

*BMI* Body Mass Index, *FIGO* the International Federation of Gynecology and Obstetrics.

**Table 2:** Clinical features of the 152 epithelial ovarian cancer (EOC) patients involved grouped by training set and validation set.

Variables	Total (n = 152)	Training group (n = 114)	Validated group (n = 38)	*p*-value
CTC counts (n/5 mL)	8.70 ± 5.69	8.64 ± 5.38	8.72 ± 4.65	0.935
M-CTC (n %)	0.24 ± 0.19	0.25 ± 0.21	0.22 ± 0.14	0.413
E-CTC (n %)	0.57 ± 0.25	0.56 ± 0.17	0.59 ± 0.23	0.392
Hybrids-CTC (n %)	0.19 ± 0.11	0.21 ± 0.18	0.20 ± 0.14	0.755
Neutrophil (10^9^/L)	5.22 ± 2.91	4.67 ± 2.83	5.31 ± 1.96	0.198
lymphocyte (10^9^/L)	1.42 ± 0.63	1.46 ± 0.85	1.37 ± 0.77	0.564
Platelet (10^9^/L)	320.39 ± 78.68	299 ± 73.94	314 ± 80.21	0.291
Albumin (g/L)	40.49 ± 6.58	38.27 ± 9.38	41.22 ± 10.02	0.101
CA-125 (U/mL)	990.71 ± 365.41	1001.23 ± 330.98	986.53 ± 310.27	0.810
CA-199 (U/mL)	129.03 ± 53.18	121.88 ± 48.27	132.5 ± 59.12	0.270
AFP (ng/mL)	6.21 ± 4.78	6.43 ± 5.99	5.93 ± 3.45	0.627
CEA (ng/mL)	3.02 ± 2.57	2.95 ± 2.60	3.21 ± 2.47	0.589
HE4 (pmol/L)	536.12 ± 54.48	542.32 ± 79.39	521.39 ± 62.10	0.141

*CTC* circulating tumor cell, *M-CTC* mesenchymal CTCs/ total CTCs percentage, *E-CTC* epithelial CTCs/ total CTCs percentage, *Hybrids-CTC* hybrids CTCs/ total CTCs percentage, *CA-125* carbohydrate antigen-125, *CA-199* carbohydrate antigen-199, *CEA* carcinoembryonic antigen, *AFP* alpha-fetoprotein, *HE4* human epididymis protein 4.

### Univariable and multivariate regression analysis of training group

**Figure 1:**
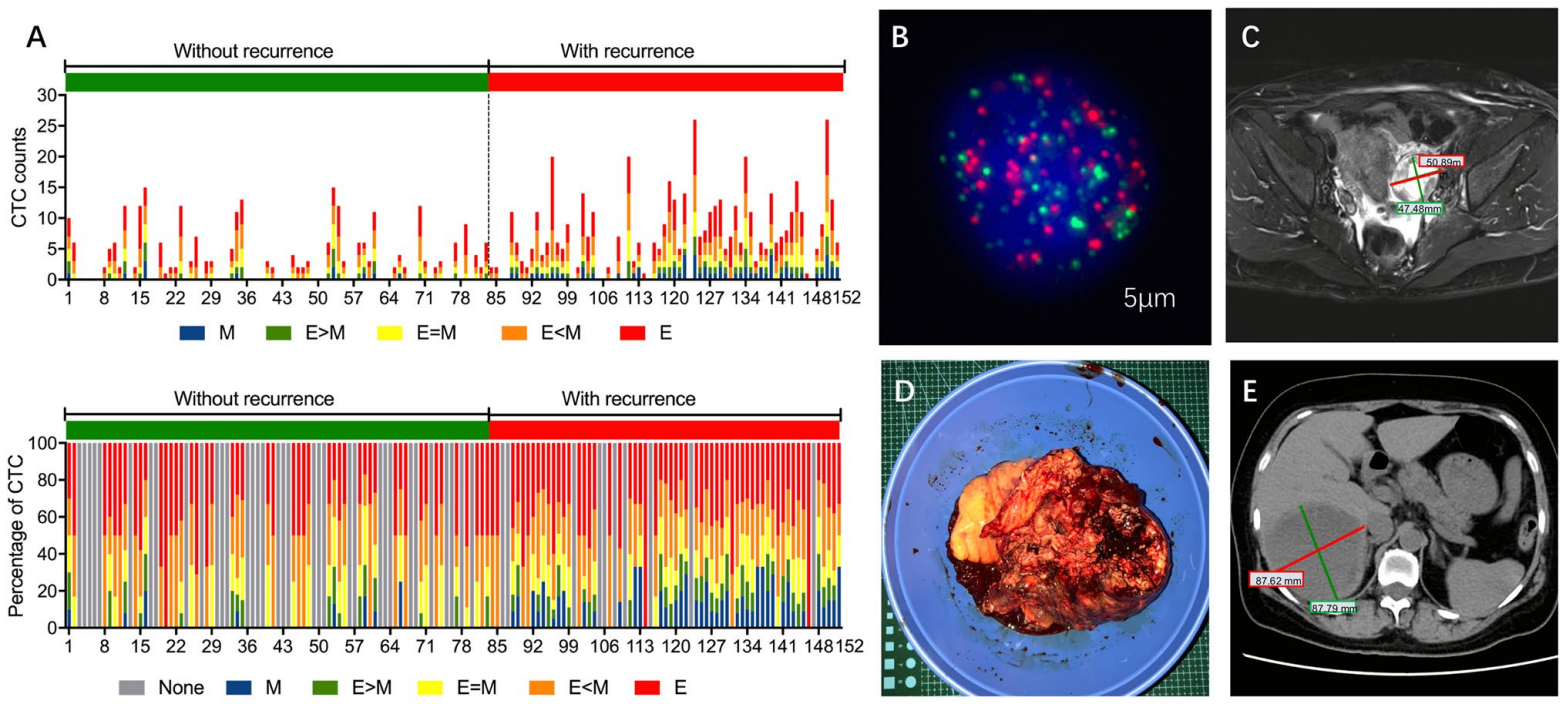
(**A**) Total CTC counts (top) and percentage of each CTC category (bottom) before treatment in ovarian cancer patients with or without recurrence. (**B**) The CTC image and (**C**) Magnetic Resonance Imaging (MRI) image of a representative patient who had the CTC-positive blood sample before treatment. (**D**) After tumor resection, the patient suffered cancer recurrence with (**E**) detectable liver metastasis observed by Magnetic Resonance Imaging (MRI) at 6-month follow-up.

Figure 1 showed that patients suffered cancer recurrence had higher CTC counts and M-CTC percentage (*p*-value < 0.05). To further determine the independent predictive indexes, univariate and multivariate analyses were performed (Table 3). In the univariable COX regression analysis, parameters including age, tumor size, menopausal status, pathological grade, FIGO stage, lymph node metastasis, ascites, CTC counts, M-CTC percentage, albumin level, CA-125 and HE4 were significantly associated with ovarian cancer recurrence. Then, these indicators were included into the multivariate Cox hazards model for further analysis. The results demonstrated that pathological grade (HR 1.382; 95% CI 1.104–3.965; *p* = 0.041), FIGO stage (HR 2.391; 95% CI 1.230–4.377; *p* = 0.011), lymph node metastasis (HR 1.312; 95% CI 1.029–2.975; *p* = 0.039), ascites (HR 1.215; 95% CI 1.067–1.806; *p* = 0.026), CTC counts (HR 1.187; 95% CI 1.098–1.752; *p* = 0.012), M-CTC percentage (HR 1.098; 95% CI 1.047–1.320; *p* = 0.009) and CA-125 (HR 1.097; 95% CI 1.021–1.373; *p* = 0.028) were independent prognostic factors for OS of EOC patients (Table 3).

**Table 3:** Clinicopathological features selection using the univariable and multivariate Cox logistic regression model among the training group.

Variables	Univariate analysis			Multivariate analysis		
	Hazard ratio	95% CI*	*p*-value	Hazard ratio	95% CI*	*p*-value
Age (years)	1.131	1.028–1.954	0.045	1.089	0.922–1.317	0.058
BMI (kg/m^2^)	1.016	0.969–1.064	0.514	–	–	–
Tumor size (cm)	1.271	1.014–1.298	0.048	0.933	0.815–1.069	0.314
**Menopausal status**						
Pre/peri-menopause	Reference	–	–	Reference	–	–
Post-menopause	1.153	1.037–1.209	0.047	3.498	0.196–6.374	0.394
**Fertility history**						
0–1	Reference	–	–	–	–	–
≥ 2	0.989	0.967–1.025	0.574	–	–	–
**Pathological grade**						
G1–2	Reference	–	–	Reference	–	–
G3	1.509	1.031–4.506	0.034	1.382	1.104–3.965	0.041
**FIGO stage**						
I–II	Reference	–	–	Reference	–	–
III–IV	2.534	1.293–4.966	0.007	2.391	1.230–4.377	0.011
**Lymph node metastasis**						
Negative	Reference	–	–	Reference	–	–
Positive	1.269	1.045–3.582	0.036	1.312	1.029–2.975	0.039
**Ascites**						
Negative	Reference	–	–	Reference	–	–
Positive	1.123	1.042–1.301	0.017	1.215	1.067–1.806	0.026
CTC counts (n/5 mL)	1.241	1.108–1.384	0.009	1.187	1.098–1.752	0.012
M-CTC (n %)	1.147	1.051–1.288	0.006	1.098	1.047–1.320	0.009
E-CTC (n %)	0.983	0.857–2.694	0.148	–	–	–
Hybrids-CTC (n %)	1.058	0.894–1.753	0.365	–	–	–
Neutrophil (10^9^/L)	1.015	0.992–1.038	0.207	–	–	–
Lymphocyte (10^9^/L)	1.066	0.888–1.278	0.494	–	–	–
Platelet (10^9^/L)	1.002	0.997–1.007	0.351	–	–	–
Albumin (g/L)	0.909	0.849–0.972	0.025	1.027	0.980–1.077	0.266
CA-125 (U/mL)	1.101	1.002–1.203	0.019	1.097	1.021–1.373	0.028
CA-199 (U/mL)	0.977	0.942–1.012	0.198	–	–	–
AFP (ng/mL)	1.001	0.963–1.040	0.958	–	–	–
CEA (ng/mL)	1.001	0.999–1.012	0.805	–	–	–
HE4 (pmol/L)	1.217	1.104–1.232	0.039	0.959	0.850–1.081	0.489

*BMI* Body Mass Index, *FIGO* the International Federation of Gynecology and Obstetrics, *CTC* circulating tumor cell, *M-CTC* mesenchymal CTCs/ total CTCs percentage, *E-CTC* epithelial CTCs/ total CTCs percentage, *Hybrids-CTC* hybrids CTCs/ total CTCs percentage, *CA-125* carbohydrate antigen-125, *CA-199* carbohydrate antigen-199, *CEA* carcinoembryonic antigen, *AFP* alpha-fetoprotein, *HE4* human epididymis protein 4.

### Construction of EOC recurrence nomogram

**Figure 2:**
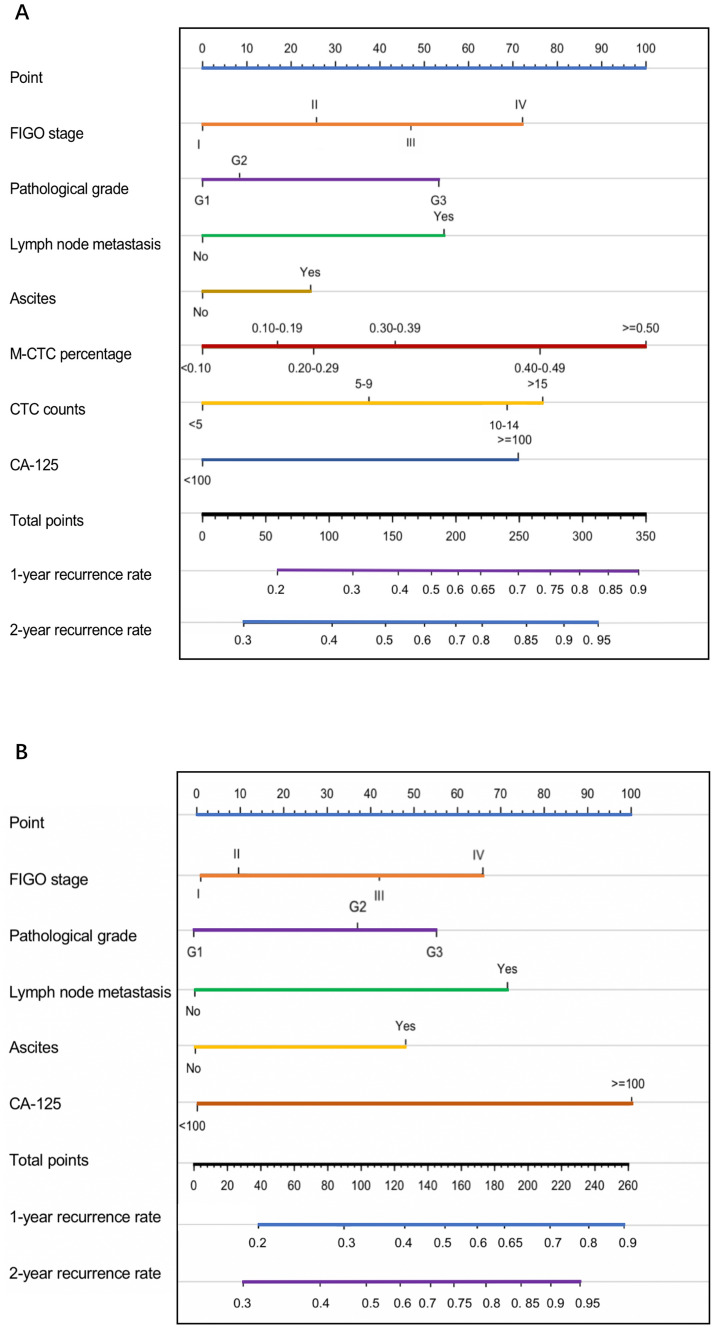
The nomogram models 1-year recurrence rate and 2-year recurrence rate of ovarian cancer patients (**A**) based on CTC counts and M-CTC percentage; (**B**) without CTC counts and M-CTC percentage. The nomogram plots were generated by the “rms” package of R software^18^.

The clinicopathological parameters (FIGO stage, pathological grade, lymph node metastasis, ascites, CTC counts, M-CTC percentage, and CA-125) selected by both univariable and multivariate Cox logistic regression were channeled into construction of the nomogram (Fig. 2A), while a nomogram without CTC counts and M-CTC percentage were also constructed for comparison (Fig. 2B). In the training group, the C-index values of 1000 sample bootstraps were 0.913 and 0.832 for the nomograms with and without CTCs. When applied to the validation cohort, the C- index values were 0.874 and 0.782, respectively, which showed a significant prognosis value of discrimination in both cohorts for the nomogram with CTC counts and M-CTC percentage.

**Figure 3:**
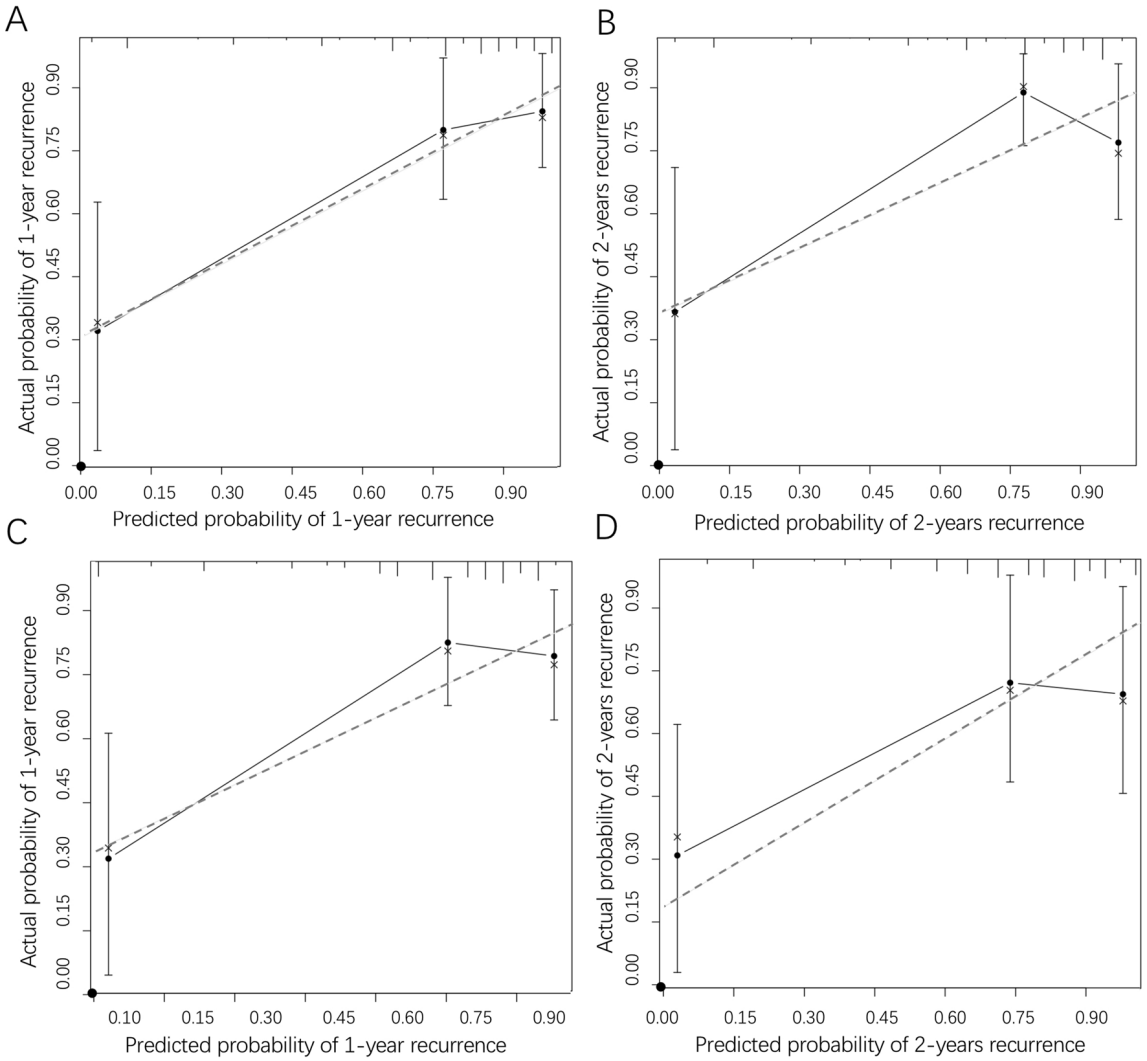
The calibration curves of internal (**A**, **B**) and external (**C**, **D**) validation of the nomogram constructed in the training group based on CTCs count and M-CTC percentage. The predicted probabilities of 1-year and 2-year recurrence were consistent with the actual recurrence proportions of ovarian cancer patients. The calibration plots were generated by the “rms” package of R software^18^.

Further risk stratification in EOC patients calibration curves manifested that the probability of predicted 1-year and 2-year recurrence rate in nomogram was well consistent between the predicted outcome of cancer recurrence and actual observation in the training group (Fig. 3A,B). Moreover, in the external validation group, the calibration curves also illustrated good validation between predicted and observed 1- and 2-year recurrence proportions (Fig. 3C,D). The discrimination and calibration validation of the external group certificated that nomogram models in this study were comparatively accurate enough to predict the recurrence probability of patients with EOC.

### Risk stratification in EOC patients

**Figure 4:**
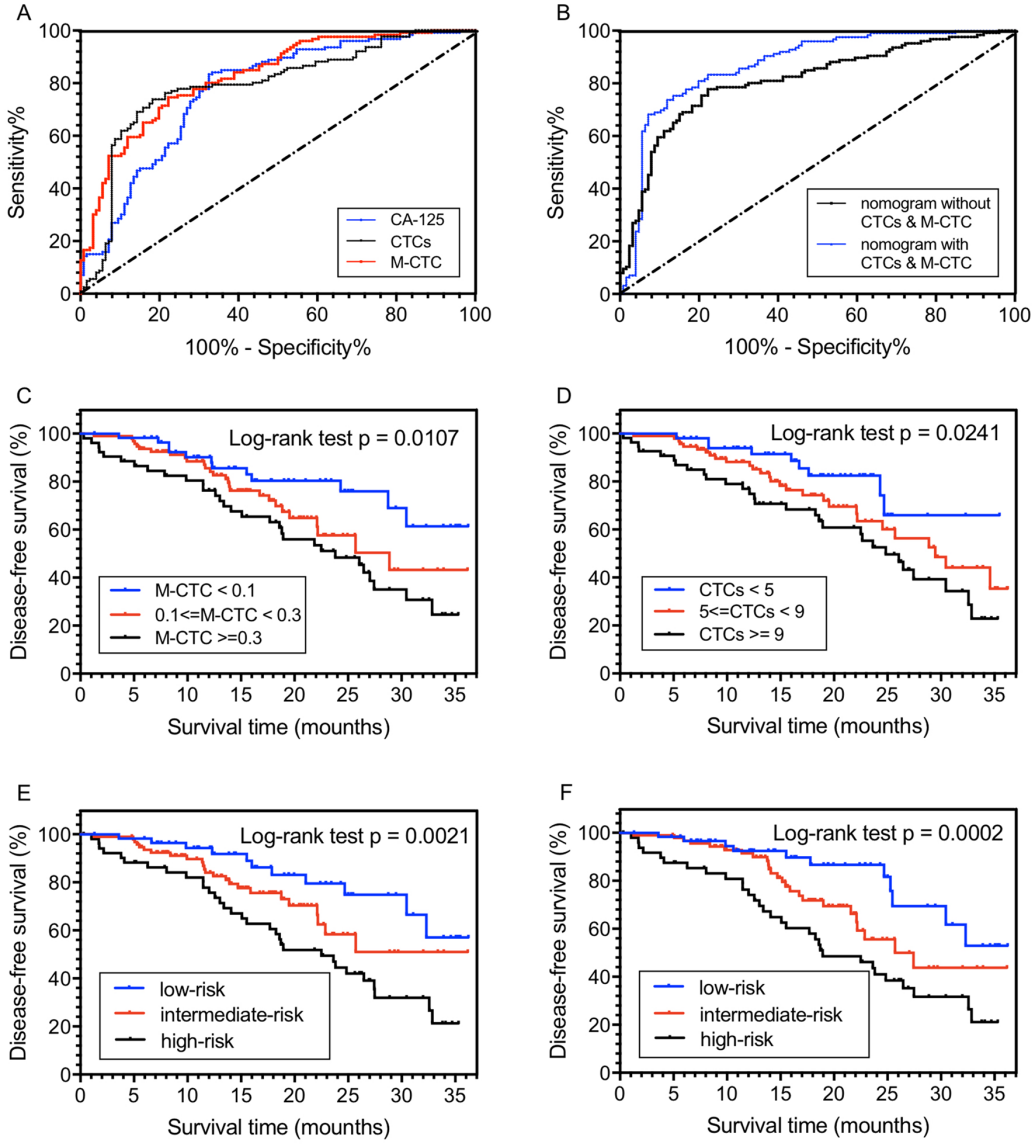
The receive operating characteristic (ROC) curve of patients stratified by (**A**) CTC counts, M-CTC percentage, and CA-125; (**B**) nomogram-based risk groups The Kaplan–Meier curves for DFS of all the patients involved stratified by (**C**) M-CTC percentage; (**D**) CTC counts and risk groups of the nomogram (**E**) without or (**F**) with CTC counts and M-CTC percentage.

According to the ROC curve, the AUC values of CTC counts, M-CTC percentage, and CA-125 were 0.8073, 0.8262, and 0.7735, respectively (Fig. 4A). For the nomogram with/without CTC counts and M-CTC percentage, the AUCs were 0.8705 and 0.8097 (Fig. 4B). Meanwhile, as illustrated in Fig. 4C,D, the discriminatory values of CTC counts and M-CTC percentage were significant among ovarian cancer patients, with the log-rank *p*-value of 0.0241 and 0.0107, respectively. When stratified by CTC counts, patients with CTCs ≥ 9 and 5 ≤ CTCs < 9 were associated with a 1.98-fold increase (95% CI 1.04–2.47) and 1.24-fold increase (95% CI 1.07–2.29) of recurrence rate, comparing to those with CTCs < 5, while patients with M-CTC percentage ≥ 0.3 and 0.1 ≤ M-CTC < 0.3 were associated with a 2.10-fold increase (95% CI 1.54–2.66) and 1.43-fold increase (95% CI 1.14–2.53) of recurrence rate, comparing to those with M-CTC < 0.1.

Moreover, the patients were then divided into three risk groups (low-, intermediate- and high-risk groups) based on the nomogram-predicted recurrence probabilities. For the nomogram without CTCs, when compared with the low-risk group, the high-risk and intermediate-risk groups were associated with a 2.37-fold increase (95% CI 1.28–4.83) and 1.48-fold increase (95% CI 1.17–2.64) in the risk of recurrence, with the *p*-value of 0.0021 (Fig. 4E). Separately, the log-rank *p*-values were 0.0386 between high and intermediate risk groups, while 0.0930 between intermediate-risk and low-risk groups. Meanwhile, in the CTCs based nomogram, the high-risk and intermediate-risk groups were associated with a 3.14-fold increase (95% CI 1.16–4.50) and 1.86-fold increase (95% CI 1.70–3.96) in the risk of recurrence, with the *p*-value of 0.0002 (Fig. 4F). Separately, the log-rank *p*-values were 0.0292 between high-risk and intermediate-risk groups, while 0.0491 between intermediate-risk and low-risk groups.

## Discussion

The clinical value of CTCs is constantly growing, as they could serve precision-medicine-based treatment of EOC patients by stratifying those with potential high recurrence risk. In this prospective study, we developed and validated a novel nomogram based on CTCs and other clinicopathological variables to categorize EOC patients concerning tumor recurrence. We also found that the presence of CTC subpopulations, especially the M-CTC percentage is associated with ovarian cancer recurrence. To our knowledge, this is the very first recurrence risk stratification developed for EOC patients especially refer to CTCs undergoing EMT.

Increasing evidence indicated that CTCs count is an independent predictor for prognosis in various solid carcinoma, including breast cancer, prostate cancer, and hepatocellular cancer. The breast cancer studies have demonstrated that patients with CTCs < 5 per 7.5 mL blood would suffer shorter PFS (2.1 months vs 7.0 months, *p* < 0.001)^19, 20^. In prostate cancer, CTCs count is considered as an independent predictor of the overall survival rate among castration-resistant prostate cancer patients (*p* < 0.05)^6^. However, regarding ovarian cancer, whether CTCs detection was associated with prognosis remains controversial^10, 21^. Judson et al.^21^ characterized CTCs by immunomagnetic beads conjugated to epithelial markers followed with the microscopic evaluation refer to specific cytoplasmic staining and did not find a significant correlation between CTCs and prognosis. In contrast, Poveda et al.^10^ analyzed CTCs using the CellSearch system and concluded that elevated CTCs could impart unfavorable prognoses of ovarian cancer patients. Differences in isolation and characterization technique in previous studies make it difficult to combine conclusions in agreement^22^. So, the standardization of CTCs detection techniques is of great importance. In our study, we revealed that CTCs count was an independent prognosis factor for ovarian cancer recurrence through both univariable and multivariable analyses using the CanPatrol CTC-enrichment technique System. The high sensitivity of the CanPatrol technique might be attributed to a simple filter-based separation method that might reduce CTC loss caused by the complicated washing and centrifugation process^23^.

Meanwhile, the routine approach of the Cellsearch System used in previous studies might fail to detect CTCs undergoing EMT, since it only isolates CTCs by the only tumor epithelial cell expression of EpCAM^11, 23^ and not mesenchymal ones without epithelial markers. Thus, we used the CanPatrol CTC-enrichment technique System to detect aggressive CTCs subpopulations that might have undergone EMT through various target sequences, including EpCAM, CD45, CK8/18/19, vimentin, and Twist^5^. For hepatocellular carcinoma, a previous study concluded that M-CTC percentage ≥ 2% before the operation was a novel predictor for early recurrence with the AUC 0.75 (95% CI 0.66–0.84)^8^, which was partly consistent with our finding that ovarian cancer patients with M-CTC percentage ≥ 0.3 and 0.1 ≤ M-CTC < 0.3 were associated with a 2.10-fold increase and 1.43-fold increase of recurrence rate, when compared to those with M-CTC < 0.1. However, regarding the results of univariable regression analysis, E-CTC percentage and hybrid-CTC percentage were not considered as independent prognostic factors for OS of EOC patients (*p*-value ≥ 0.05). To the best of our knowledge, this is the first study to reveal the considerable clinical value of both CTC counts and M-CTC percentage in ovarian cancer prognosis.

Moreover, we aimed to develop a predictive nomogram to help facilitate the risk triage of ovarian cancer recurrence. Besides the presence of CTCs, we also selected several routinely collected risk factors including pathological grade, FIGO stage, lymph node metastasis, ascites, and CA-125 to construct the nomogram in training group^24–26^. The clinical relevance of our nomogram was demonstrated by its internal and external validation with the C-index of 0.913 and 0.874, which indicated that our model included in CTCs could provide a more reliable predictive evaluation for ovarian cancer recurrence than previous studies^26, 27^.

Nevertheless, we further performed risk stratification of EOC patients based on CTC counts, M-CTC percentage, and points derived from the nomogram. All the risk stratification was well validated by survival analysis (*p* < 0.05) with the AUC higher than 0.75 as well. According to risk stratification, especially by the nomogram, we could carry out individualized and targeted treatment to improve the prognosis of ovarian cancer.

However, there are also some limitations of our study. Firstly, the prospective study enrolled a relatively small sample size of 152 EOC patients in a single-center, which might limit the accuracy of results. To overcome this problem, additional multi-center studies with a larger sample size would be of great importance to further validate our results. Second, detection efficiency might be biased since the CanPatrol system is a filtration-based system, allowing small CTCs to easily cross the barrier. Thus, other CTCs collection techniques might also be used to improve detection efficiency in future studies.

In conclusion, CTCs, especially those undergoing EMT hold promise prognostic value as minimally-invasive biomarkers for ovarian cancer recurrence. By the advanced CanPatrol CTC-enrichment technique, our study evaluated both CTC counts and M-CTC percentage to clarify their clinical value. The prognostic nomogram based on CTCs and EMT could support clinical decision-making and provide cues for early intervention among EOC patients.

## Methods

### Study design and patients

**Figure 5:**
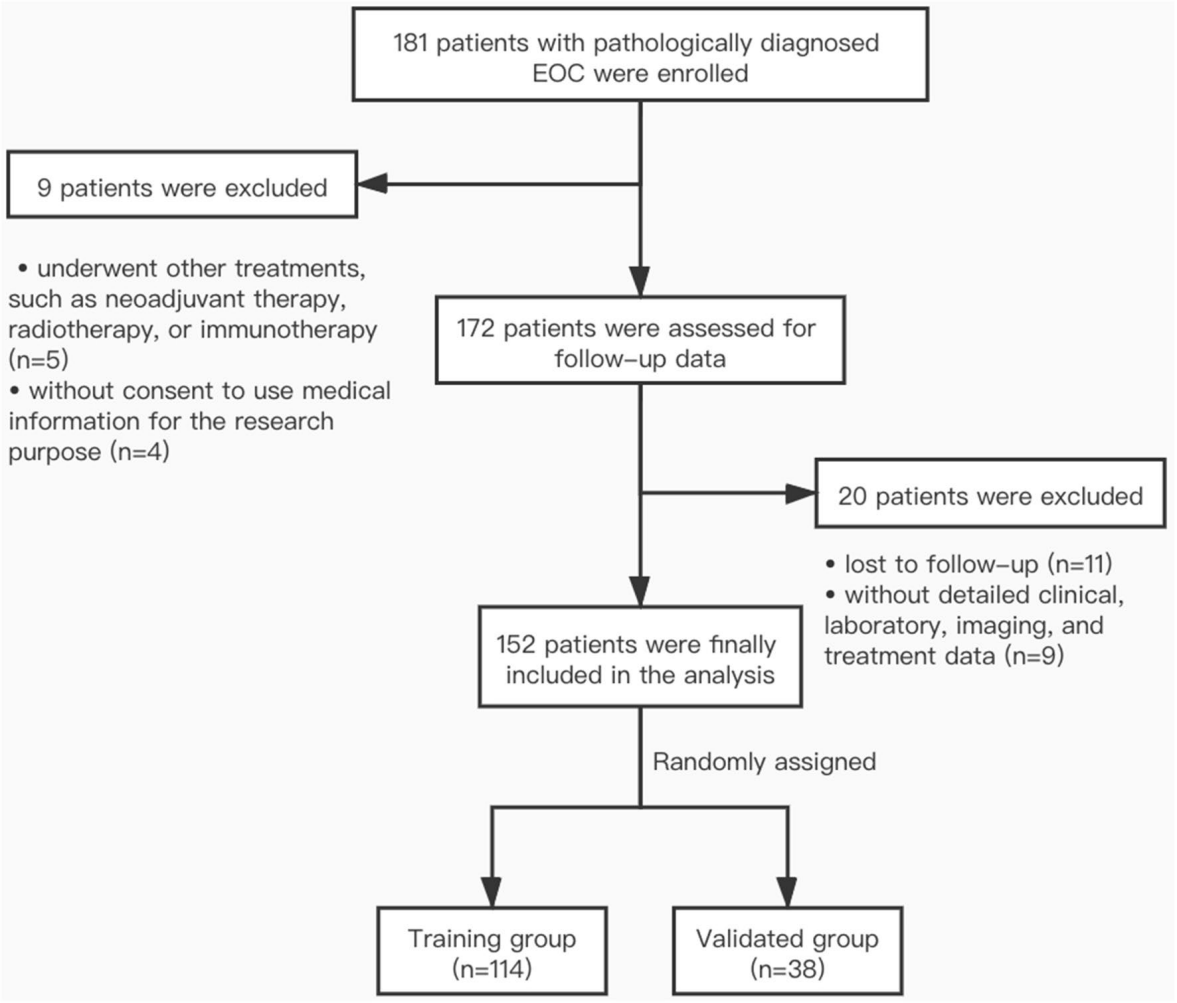
EOC Patient enrollment flow chart.

We enrolled 181 patients with pathologically diagnosed EOC who underwent surgery at the Department of Obstetrics and Gynecology, Renji Hospital Affiliated to Shanghai Jiaotong University School of Medicine between June 2017 to October 2019. The criteria for inclusion in this study were: (1) newly diagnosis EOC confirmed by pathological biopsy; (2) no coexisting cancers or prior cancers within 5 years; (3) no preoperative treatment, including neoadjuvant chemotherapy or radiotherapy. The exclusion criteria were as follows: (1) lost to follow-up (n = 9); (2) without detailed clinical, laboratory, imaging, and treatment data (n = 8); (3) underwent other treatments, such as radiotherapy or immunotherapy (n = 5); (4) without consent to use medical information for the research purpose (n = 4), and (5) with status not allowing the treatment of operation followed by chemotherapy (n = 3). As a result, 152 patients were assessed in the analysis (Fig. 5). Moreover, we also involved 30 patients with benign gynecologic diseases at our institution as negative controls.

In order to achieve optimal tumor debulking, the operation for all involved patients was aimed at maximal ovarian tumor resection without visible residual tumor. The surgery was followed by standardized paclitaxel and platinum chemotherapy. All patients were followed up until September 1st, 2020. This study was approved by the Ethics Committee of Renji Hospital Affiliated to Shanghai Jiaotong University School of Medicine and all involved subjects provided informed consent for use of their information for research purposes. All experiments were performed in accordance with relevant guidelines and regulations.

### Clinicopathological data collection

The clinical stage was evaluated according to the International Federation of Gynecology and Obstetrics (FIGO) stage system. Routine blood tests and tumor marker measurements, including carbohydrate antigen-125 (CA-125), carbohydrate antigen-199 (CA-199), carcinoembryonic antigen (CEA), alpha-fetoprotein (AFP), and human epididymis protein 4 (HE4) were conducted within 1 day before surgery. The clinicopathologic variables, including age, Body Mass Index (BMI), tumor size, menopausal status, fertility history, pathological grade, the FIGO stage, lymph node metastasis, ascites, and histological type were reviewed from medical records. Disease-free survival (DFS) was measured from the date of surgery to the last follow-up visit or ovarian cancer recurrence, which was defined through the latest clinical evidence. The diagnosis of EOC recurrence was performed by at least two oncologists to avoid bias.

### Isolation and characterization of CTCs

Peripheral blood samples (5 mL, anticoagulated with EDTA) were collected 1 day before treatment, stored at 2–8℃, and processed within 4 h after sampling^7^. To avoid potential skin cell contamination caused by venipuncture, the first 2 mL of blood was discarded^28^.

**Figure 6:**
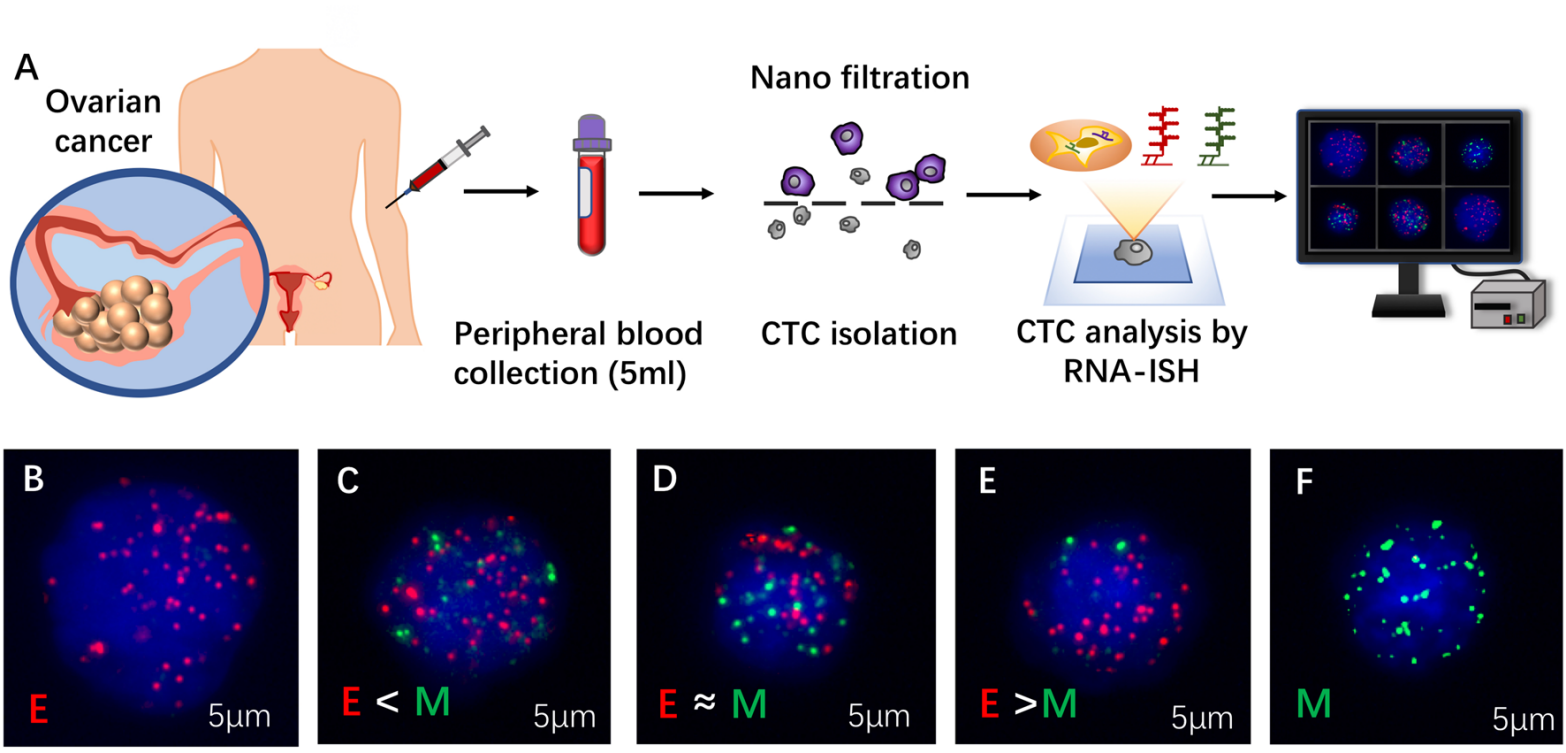
(**A**) Process of circulating tumor cells (CTCs) isolation and detection by CanPatrol CTC enrichment and ISH. (**B**–**F**) 5 representative images of patients with different CTC subpopulations, based on the RNA-ISH of mesenchymal (vimentin and Twist, green fluorescence) and epithelial (EpCAM and CK8/18/19, red fluorescence) markers.

In this study, we isolated and characterized CTCs through the CanPatrol system (Fig. 6). Firstly, the blood sample preserved in cell preservation solution was centrifuged for 5 min at a speed of 1850 rpm. After removing the supernatant, the sample was mixed with phosphate buffer saline (PBS) and 4% formaldehyde for 8 minutes^7^. For filtration, we passed the sample through the vacuum filtration system at 0.08 MPa^7^.This system included a filtration tube containing the membrane with 8-μm diameter pores, a vacuum pump, and a manifold vacuum plate with valve settings.

RNA-ISH was used to detect CTCs through the following target sequences: CD45 (leukocyte biomarker); vimentin and Twist (mesenchymal biomarkers); EpCAM and CK8/18/19 (epithelial biomarkers)^8, 29, 30^ (Fig. 6). The amplification process was performed on a 24-well plate. Then, we treated the samples with protease K and hybridized the cells with fluorescent probes specific for target sequences: red for epithelial cell adhesion molecule (EpCAM and CK8/18/19) and green for the mesenchymal molecule (Vimentin and Twist)^31^. We used 40,6-diamidino-2- phenylindole (DAPI) to stain the nuclei, and the cells were analyzed with a fluorescent microscope (Olympus Corporation, Tokyo, Japan)^12^. Based on these markers, we then classified CTCs into three subgroups: epithelial (epithelial markers+/mesenchymal markers−/CD45−/DAPI+), mesenchymal (epithelial markers−/mesenchymal markers+/CD45−/DAPI+), and hybrids (epithelial markers+/mesenchymal markers+/CD45−/DAPI+).

### Construction of nomogram

The dataset was randomly divided into training and validation cohorts. The selection bias refer to the random classification of the two cohorts was adjusted^32^. T-test and Chi-square test were used to analyze the differences of clinicopathologic characteristics between two cohorts for continuous and categorical variables, respectively. The prognostic factors were determined using both univariate and multivariate analyses through Cox's hazards regression model. The nomogram and calibration plots were generated with the “rms” package of R software^18^. Nomogram points, ranging from 1 to 100, were assigned refer to the weights for the relative importance of each model covariate determined by the final hazards regression model. In the nomogram, the total score for each patient was evaluated as a weighted sum of the contribution from each risk factor to predict the probability of recurrence at 1 and 2 years.

### Validation of nomogram

The predictive ability of the nomogram model was measured by both discrimination and calibration. The discrimination of the nomogram model was evaluated by calibration curves, overlaying the observed probabilities and nomogram-predicted probabilities with 95% confidence interval (95% CI). As a measurement for internal validation, the Harrell's concordance index (C-index) was analyzed using tenfold cross-validation repeated for 20 times^33^. The calibration plots were generated by the “rms” package of R software^18^.

We categorized patients into three risk groups of CTC counts, M-CTC percentage, and nomogram, based on the X-tile (Version 3.6.1, Yale University, New Haven, USA), a newly-developed bioinformatic tool to determine optimal cut-off points for survival analysis^34^. The X-tile software could test all possible cut-off points of target quantitative data by Log-rank test and selected the lowest *p*-value and highest χ2. The EOC patients involved were then divided into three risk groups: good, intermediate, and poor prognosis. The optimal cut-off values were 128 and 251 for the nomogram with CTCs, while 98 and 169 for the nomogram without CTCs. Taking CTCs into separation, the values were 5 and 9 in CTC count, 0.1 and 0.3 in the M-CTC percentage. Kaplan–Meier methods were used to generate the survival curves and the prognostic differences were assessed by Log-rank test. The receiver operating characteristic (ROC) curve analysis was applied to identify the prognosis value of the nomogram according to the Youden index and area under the curve (AUC). All the statistical analyses were conducted by R software Version 4.0.2 (GUI 1.72 Catalina build, https://www.R-project.org) and graphed using Graph Prism Version 7.0a (GraphPad Software, San Diego, CA, USA). *p*-value < 0.05 was defined as statistically significant.

## Data Availability

The data of these findings cannot be shared at this time as the data also forms part of an ongoing study. Requests for data will be considered by the corresponding author after publication of the study.
